# Cardiovascular risk factors and life expectancy in India: A retrospective study

**DOI:** 10.6026/973206300213242

**Published:** 2025-09-30

**Authors:** Prashant Kumar, Venkata N. Bhavani Penmathsa, Daisy Bacchani, Rahul Tiwari, Heena Dixit Tiwari, Prashant M.C., Deepak Sharma, Tohid Ali

**Affiliations:** 1 Department of Cardiology, Rajendra Institute of Medical Sciences, Ranchi, Jharkhand, India; 2 Dr Pinnamaneni Siddhartha Institute of Medical Sciences and Research Foundation (PSIMS and RF), Vijayawada, Andhra Pradesh, India; 3 Department of Microbiology, Vyas Medical College and Super Speciality Hospital, Jodhpur, Rajasthan, India; 4 Department of Oral and Maxillofacial Surgery, RKDF Dental College and Research Centre, Sarvepalli Radhakrishnan University, Bhopal, Madhya Pradesh, India; 5 Department of Medical Health Administration, Index Institute, Malwanchal University, Index City, Nemawar Road, Indore, Madhya Pradesh, India

**Keywords:** Cardiovascular disease, risk factors, life expectancy, India, retrospective cohort, survival analysis

## Abstract

Cardiovascular disease (CVD) remains the leading cause of adult mortality in India. Hence, we retrospectively analyzed electronic records of 4,812 adults from two tertiary hospitals to quantify major CVD risk factors and their association with survival and estimated remaining life expectancy (LE) at age 40 using Chiang's abridged life table with Sample Registration System (SRS) background rates. Hypertension, diabetes, dyslipidemia, smoking and obesity were frequent (≥20% each). Adjusted Cox models showed stepwise higher mortality with increasing risk-factor count (≥3 vs 0: HR 1.92; 95% CI 1.49-2.48; p<0.001). Estimated LE at 40 declined from 39.6 (0 factors) to 33.2 years (≥3). Findings underscore intensified primary prevention.

## Background:

India faces a substantial and growing burden of cardiovascular disease (CVD) with ischaemic heart disease and stroke among the top causes of disability-adjusted life years (DALYs) in 2019 [1]. Exposure to modifiable risk factors-elevated blood pressure, hyperglycemia, dyslipidemia, smoking and high body mass index-accounts for a large share of CVD burden in Global Burden of Disease (GBD) assessments [2]. Indian data document high and heterogeneous prevalence of hypertension (≈21-24% in adults; higher in urban areas) and suboptimal control [3, 4], widespread diabetes with marked state-level variation [5] and pervasive dyslipidemia (low HDL-C, hypertriglyceridemia) [6, 7]. Environmental exposures such as ambient PM2.5 further aggravate cardiometabolic risk and mortality [8, 9]. Concurrently, national life expectancy (LE) gains slowed and partially reversed during 2017-2021, with period LE at birth estimated at 69.8 years for 2017-2021 and declines during the COVID-19 pandemic [10, 11 and 12]. Despite abundant risk-factor surveillance, fewer hospital-based Indian studies directly relate cumulative risk-factor burden to mortality and LE at the patient level using indigenous background mortality schedules. Therefore, it is of interest to describe the prevalence of major CVD risk factors in Indian tertiary-care attendees, quantify their association with mortality and show corresponding differences in remaining LE at age 40.

## Materials and Methods:

## Design, setting, participants:

We performed a retrospective cohort study of adults' ≥30 years attending two tertiary hospitals. Electronic health records were linked to hospital vital status. We randomly sampled one index encounter per person. Exclusions: known CVD at baseline (IHD, stroke), cancer, CKD stage ≥4, or missing key covariates. Final analytic sample: n=4,812.

## Variables:

Risk factors at baseline: hypertension (SBP ≥140/DBP ≥90 or antihypertensive use), diabetes (FPG ≥126 mg/dL or HbA1c ≥6.5% or medication), dyslipidemia (LDL-C ≥130 mg/dL or HDL-C <40 men/<50 women or TG ≥150 mg/dL or lipid therapy), current smoking and obesity (BMI ≥30 kg/m^2^). We created a risk-factor count (0, 1, 2, ≥3). Covariates: age, sex, residence (urban/rural) and hospital.

## Outcomes and analysis:

Primary outcome: all-cause mortality. We used Kaplan-Meier curves and Cox proportional hazards models adjusted for age, sex, residence and hospital. Proportional hazards were checked via Schoenfeld residuals. For LE, we applied Chiang's abridged life table using SRS 2017-2021 mortality as baseline and incorporated adjusted hazard ratios to derive risk-stratified mortality schedules at age 40, estimating remaining LE. Two-sided α=0.05. Analyses were conducted in R 4.3.

## Results:

Mean age was 52.1±11.9 years; 51.4% were men; 68% urban. Prevalence was: hypertension 28.7%, diabetes 21.9%, dyslipidemia 44.8%, current smoking 23.1% and obesity 22.6%. Overall, 18.9% had 0 risk factors, 30.4% had 1, 28.1% had 2 and 22.6% had ≥3. Median follow-up was 6.8 years (IQR 4.0-9.1). There were 462 deaths (9.6%) (Table 1 - see PDF). In adjusted Cox models, mortality increased with each additional risk factor (p-trend <0.001). Compared with 0 factors, HRs was: 1 factor 1.28 (95% CI 0.98-1.68; p=0.07), 2 factors 1.57 (1.22-2.02; p<0.001), ≥3 1.92 (1.49-2.48; p<0.001). Individual risk factors associated with mortality were hypertension (HR 1.31; 1.09-1.58; p=0.004), diabetes (1.29; 1.05-1.59; p=0.016), smoking (1.37; 1.12-1.68; p=0.002) and obesity (1.18; 0.95-1.46; p=0.13). Dyslipidemia showed a modest association (1.14; 0.94-1.39; p=0.18). Estimated remaining LE at age 40 declined from 39.6 years with 0 factors to 37.9 (1 factor), 35.8 (2 factors) and 33.2 (≥3 factors); difference between ≥3 and 0 = -6.4 years. The LE gradient persisted in sex-stratified analyses (p-interaction=0.21) (Table 2 - see PDF).

## Discussion:

In this multi-site retrospective cohort, major CVD risk factors were highly prevalent and clustered: nearly one in four adults presented with three or more. The prevalence levels we observed align with national surveillance and prior literature. Hypertension in our cohort (28.7%) sits within the range reported for India by NFHS-5 and systematic reviews (≈21-24%) and is consistent with urban-rural and sex differences documented previously [3, 4]. Diabetes prevalence (21.9%) parallels estimates from ICMR-INDIAB, which reported wide state variation and an urban excess [5]. Our dyslipidemia burden (44.8%) reflects the Indian pattern of atherogenic dyslipidemia dominated by low HDL-C and hypertriglyceridemia, as described by Joshi *et al.* and in updated trend analyses [6, 7]. The high smoking prevalence among men is congruent with national data and helps explain sex differences in CVD outcomes. Crucially, we demonstrate a graded association between cumulative risk-factor count and mortality, with nearly two-fold higher adjusted hazards in individuals with ≥3 factors compared with none. This stepwise pattern mirrors GBD findings attributing substantial CVD burden to joint exposure to metabolic and behavioral risks [2]. Our life-expectancy modeling translates these hazards into interpretable population metrics: at age 40, those with ≥3 risk factors are estimated to live ~6.4 fewer years than peers with none, even when anchored to Indian background mortality from SRS. While GBD and national reports have tracked slowing LE gains and pandemic-related declines [10, 11-12], patient-level demonstrations of LE gradients by risk-factor clustering in Indian clinical settings have been sparse.

Environmental context is relevant. Evidence from the India State-Level Disease Burden Initiative and recent time-series analyses links ambient PM_2.5_ to substantial mortality and cardiometabolic risk, including diabetes [8, 9]. Our data did not directly measure pollution exposure, but given city locations (Mumbai, Bengaluru), ambient exposures likely contributed to the observed risk milieu. Integrating air-quality mitigation with classical CVD prevention could therefore amplify gains. Strengths include a sizable, contemporary cohort and the use of Indian mortality schedules for LE estimation. Limitations include hospital-based sampling (reducing generalizability), potential misclassification of risk factors based on routine records, residual confounding (socioeconomic status, diet, physical activity) and lack of cause-specific mortality. Our life-table approach assumes proportional scaling of mortality hazards across ages, which may over- or under-estimate LE differences if hazards vary by age. Nonetheless, the consistency of our risk-gradient with national and global literature supports the validity of the inferences [13, 14-15]. Overall, intensify detection and control of hypertension and diabetes, smoking cessation, lipid management and weight control-paired with air-quality interventions-are likely to deliver meaningful survival and life-expectancy benefits in Indian adults.

## Conclusion:

Among 4,812 Indian adults without baseline CVD, cardiometabolic risk factors were common and clustered. Mortality increased stepwise with risk-factor count and remaining life expectancy at age 40 was ~6.4 years lower for individuals with ≥3 risk factors versus none. Hence, health systems should prioritize integrated risk-factor management to improve survival and extend healthy life years in India.
